# Error in Author Affiliation

**DOI:** 10.1001/jamanetworkopen.2020.20371

**Published:** 2020-08-25

In the Invited Commentary titled “Reopening Colleges During the Coronavirus Disease 2019 (COVID-19) Pandemic—One Size Does Not Fit All,”^1^ published July 31, 2020, there was an error in the author affiliations. Dr Fox is located in Arlington, Virginia. This article has been corrected.^1^
